# Reach Outcomes and Costs of Different Physician Referral Strategies for a Weight Management Program Among Rural Primary Care Patients: Type 3 Hybrid Effectiveness-Implementation Trial

**DOI:** 10.2196/28622

**Published:** 2021-10-20

**Authors:** Gwenndolyn Porter, Tzeyu L Michaud, Robert J Schwab, Jennie L Hill, Paul A Estabrooks

**Affiliations:** 1 Department of Health Promotion University of Nebraska Medical Center Omaha, NE United States; 2 Center for Reducing Health Disparities University of Nebraska Medical Center Omaha, NE United States; 3 University of Nebraska Medical Center Omaha, NE United States; 4 Department of Population Health Sciences University of Utah Salt Lake City, UT United States

**Keywords:** weight management, rural, RE-AIM, hybrid effectiveness-implementation, primary care, obesity, physicians, digital health, health technology, mobile phone

## Abstract

**Background:**

Rural residents are at high risk for obesity; however, little resources exist to address this disproportional burden of disease. Primary care may provide an opportunity to connect primary care patients with overweight and obesity to effective weight management programming.

**Objective:**

The purpose of this study is to examine the utility of different physician referral and engagement processes for improving the reach of an evidence-based and technology-delivered weight management program with counseling support for rural primary care patients.

**Methods:**

A total of 5 rural primary care physicians were randomly assigned a sequence of four referral strategies: point-of-care (POC) referral with active telephone follow-up (ATF); POC referral, no ATF; a population health registry–derived letter referral with ATF; and letter referral, no ATF. For registry-derived referrals, physicians screened a list of patients with BMI ≥25 and approved patients for participation to receive a personalized referral letter via mail.

**Results:**

Out of a potential 991 referrals, 573 (57.8%) referrals were made over 16 weeks, and 98 (9.9%) patients were enrolled in the program (58/98, 59.2% female). Differences based on letter (485/991, 48.9%) versus POC (506/991, 51.1%) referrals were identified for completion (100% vs 7%; *P<*.001) and for proportion screened (36% vs 12%; *P<*.001) but not for proportion enrolled (12% vs 8%; *P=*.10). Patients receiving ATF were more likely to be screened (47% vs 7%; *P<*.001) and enrolled (15% vs 7%; *P<*.001) than those not receiving ATF. On the basis of the number of referrals made in each condition, we found variations in the proportion and number of enrollees (POC with ATF: 27/190, 50%; POC no ATF: 14/316, 41%; letter ATF: 30/199; 15.1%; letter no ATF: 27/286, 9.4%). Across all conditions, participants were representative of the racial and ethnic characteristics of the region (60% female, *P=*.15; 94% White individuals, *P=*.60; 94% non-Hispanic, *P=*.19). Recruitment costs totaled US $6192, and the overall recruitment cost per enrolled participant was US $63. Cost per enrolled participant ranged from POC with ATF (US $47), registry-derived letter without ATF (US $52), and POC without ATF (US $56) to registry-derived letter with ATF (US $91).

**Conclusions:**

Letter referral with ATF appears to be the best option for enrolling a large number of patients in a digitally delivered weight management program; however, POC with ATF and letters without ATF yielded similar numbers at a lower cost. The best referral option is likely dependent on the best fit with clinical resources.

**Trial Registration:**

ClinicalTrials.gov NCT03690557; http://clinicaltrials.gov/ct2/show/NCT03690557

## Introduction

### Background

Obesity is a pressing health concern nationwide, particularly in small rural communities. Rural residents are more obese, on average, than their urban counterparts [1] and often have no or limited access to obesity prevention and treatment programming [1,2]. Furthermore, individuals who use primary care are proportionally more obese than the general public [3], highlighting rural primary care patients as a high-need population regarding weight management. Primary care providers are often the only resource to support healthful eating, physical activity, and weight management in rural communities [4]. Primary care systems may offer a practical and sustainable venue for implementing evidence-based weight management interventions; however, little is known about how rural primary care physicians can pragmatically refer and enroll a large and representative group of individuals into an evidence-based weight management program.

A challenge for weight management interventions is ensuring that not only is an intervention effective but it also has the potential to reach populations at risk that could most benefit [5]. Factors beyond program effectiveness and total sample size, such as proportional yield from those recruited and sample representativeness, are important indicators of an intervention’s impact [6]. Few studies provide a comprehensive report on the methods used to recruit participants, and even fewer report on the representativeness of the sample when compared with the target population [7,8]. In a systematic review of rural weight loss interventions, only 2 of 53 studies compared the demographic characteristics of the intervention sample with those of the target population [8]. In a 2016 systematic review of recruitment strategies for young adult weight gain prevention interventions, 23 of 25 studies were reported to have insufficiently described the recruitment process [7]. Documenting recruitment methods and the representativeness outcomes of those efforts can lead to a better overall understanding of how best to engage patients and maximize the reach of evidence-based behavioral interventions. This understanding is important for scale-up efforts, for preventing underrepresentation of populations experiencing disparities in research and in health care, and for physicians to improve their standard of care [9].

However, when drawing more broadly from the behavioral intervention literature, a number of examples of both active and passive recruitment strategies can be found that have been used to engage various target populations [7,10-13]. On the basis of the available literature, active recruitment strategies—those with direct interaction with potential participants, such as outreach telephone calls—appear to yield a lower absolute number of participants but a higher proportion of those exposed to recruitment strategies when compared with passive recruitment strategies (ie, those without direct interaction with potential participants, such as flyers or targeted mailings), which yield a higher number but a lower proportion of participants [5]. Active recruitment strategies may also yield a more representative sample than passive recruitment strategies [12,14,15]. The limited knowledge in this area warrants further study.

### Objectives

The purpose of this study is to examine the utility and cost of different physician referral and engagement processes for improving reach (ie, number, proportion, and representativeness of participants) for a rural, evidence-based, and technology-delivered weight management program with counseling support. Our design is based on feedback gathered from prior qualitative work [16,17]; that is, an expressed clinical interest in testing the relative reach of engaging patients at the point of care (POC) during a well or chronic care visit or proactively reaching out to patients using a population health management approach facilitated by an electronic health record.

We use the RE-AIM (Reach, Effectiveness, Adoption, Implementation, and Maintenance) framework to guide our assessment of reach [18] and hypothesize that active recruitment strategies would yield a more representative sample than passive recruitment strategies. Reach refers to the absolute number, proportion, and representativeness of the participants compared with those who were exposed to recruitment efforts [18]. We also hypothesize that POC referrals, compared with the population health registry–derived letter referrals, would yield a higher proportion of enrolled participants from those who received a referral.

## Methods

### Intervention Selection

Before this pilot trial, we conducted focus groups with primary care staff employed at rural primary care clinics regarding the feasibility of implementing a weight loss program through primary care. Overwhelmingly, primary care staff agreed that a program to which physicians and nurses could refer eligible patients and track their progress throughout was more favorable than a program that would require physician- or nurse-led delivery [16,17]. We then assembled primary care physicians, staff, and obesity treatment experts from the Great Plains Practice-Based Research Network to engage in a participatory selection process [19] to identify and, if necessary, adapt an evidence-based intervention to pilot-test through a rural primary care clinic. The selection process is described elsewhere [17]. Ultimately, a digitally delivered intervention was selected by the group for local testing, and it was agreed that testing potential recruitment strategies was a priority over testing the relative effectiveness of the different weight loss programs.

### Study Design

#### Overview

The study design used the hybrid methodologies described by Curran et al [20], which allow for blended design components targeting effectiveness and implementation but focus on systems-based approaches to improve dissemination at the participant level rather than implementing the evidence-based intervention. As such, we classified this trial as a hybrid type 3 effectiveness-implementation trial focused on dissemination at the participant level (ie, reach). This allowed us to test the utility of different dissemination strategies to increase program reach as a primary outcome while concurrently gathering information on intervention effectiveness [20]. We partnered with a rural primary care clinic and the 5 physicians serving their patients to implement an evidence-based and digitally delivered weight loss program. The 5 physicians were randomly assigned to a sequence of four referral strategies over a span of 16 weeks: POC referral with active telephone follow-up (ATF); POC referral, no ATF; a population health registry–derived letter referral with ATF; and letter referral, no ATF.

#### Dissemination Strategies: Referral Methods

Referral strategies varied by POC versus a population health electronic health record–derived letter referral and ATF versus no telephone follow-up. For POC referral, physicians were instructed to refer any adult patient to an evidence-based weight loss program with diet and physical activity counseling based on (1) BMI≥25 kg/m^2^, (2) no contraindications to participation, and (3) visiting the clinic for a chronic care or well visit. In the population health approach, a clinic administrator pulled a list of patients from the electronic health record system with BMI≥25 who had visited the clinic in the previous 2 weeks. Each physician reviewed this list and removed any patients with contraindications to participation in the evidence-based weight loss program. The remaining patients were mailed a personalized invitation letter to participate in the weight loss intervention that was signed by their physician.

For ATF, the referred patient was informed that they would be contacted by a member of the research team to determine if they would like to participate in the program (an opt-out telephone number was also provided). In conditions without ATF, the referred patient was provided a telephone number to call if they were interested in discussing participation in the program. The study was reviewed and approved by the University of Nebraska Medical Center Institutional Review Board (#581-18-EP).

We designed this pilot study to recruit over a 16-week period, with the goal of enrolling approximately 100 participants and projected a 10% enrollment rate based on the overall denominator of potential referrals based on prior work [21,22]. Each physician (n=5) was randomly assigned a sequence of the four referral strategies, shifting strategies every 2 weeks (Table 1). Over the course of 16 weeks, physicians used each of the four referral strategies twice to eliminate any potential time effect on a single strategy. Each physician’s referral strategy sequence was randomized to prevent any order effect on the yield of patients per referral strategy. A member of the research team visited the clinic every 2 weeks to remind physicians of their new referral strategy; a nurse at the clinic monitored the physicians daily to ensure that the referral strategies were implemented with fidelity.

**Table 1:** Randomization sequence of referral strategy by physician.

Weeks	Physician A	Physician B	Physician C	Physician D	Physician E
1-2	L^a^	POC+^b^	POC^c^	L+^d^	POC+
3-4	POC+	POC	L+	L	L+
5-6	L+	L+	L	POC	L
7-8	POC	L	POC+	POC+	POC
9-10	L+	POC+	L+	POC	L
11-12	L	POC	POC+	L	L+
13-14	POC+	L+	L	L+	POC
15-16	POC	L	POC	POC+	POC+

^a^L: letter referral without active telephone follow-up.

^b^POC+: point-of-care referral with active telephone follow-up.

^c^POC: point-of-care referral without active telephone follow-up.

^d^L+: letter referral with active telephone follow-up.

### Enrollment

All referred and interested patients were required to undergo a brief telephone screening with a member of the research team and, if eligible, were given instructions over the phone and via email on how to complete web-based program enrollment. In addition, all interested and eligible patients were scheduled for an in-person enrollment visit with a member of the research team where they were given intervention program materials (ie, home scale) and a tutorial of the intervention’s mobile app, provided with tips on how to stay engaged with the program, and completed the web-based program enrollment (if not already complete). Patient consent to participate in the weight loss program was obtained during the web-based enrollment process. Patients were given the opportunity to raise questions before consent and during the in-person enrollment visit.

### Evidence-Based Weight Loss Program

All referred patients were offered a 12-month, digitally delivered, evidence-based weight management program free of charge. The program featured a social cognitive theory–based curriculum with counseling support delivered through daily emails and text messages. Program features also included daily meal plans and physical activity recommendations [23]. Estabrooks et al [23] provided a comprehensive overview of the program. In addition, modest financial incentives were offered with the intent to increase program reach and retention [24]. Incentives were offered to participants who lost a minimum of 5% of their initial body weight (US $15/quarter reward) graded up to a maximum of 30% body weight reduction (US $150/quarter reward). Program participants were provided a Bluetooth-enabled home scale (Smart Scale, incentaHEALTH, LLC), which connected to the program smartphone app that was installed on their smartphones during the enrollment visit. Participants were instructed to record their weight using this scale no less than once per quarter. The program also featured a website where participants could receive feedback on their weigh-ins, take health quizzes, and self-assess their progress with regard to healthy eating, physical activity, and weight loss. Upon completion of the program, participants could keep the home scale. This paper focuses on reach, which is the primary outcome of this study; data on weight loss are not presented here.

### Data Analysis

Program reach was measured by the number and proportion of individuals who were (1) referred, (2) screened, and (3) enrolled and was compared across referral strategies relative to the number of eligible patients who visited the clinic during the recruitment period. Representativeness of the enrolled sample was assessed relative to the demographic characteristics of the region, as measured by the US Census [25]. We used chi-square tests to examine group differences in terms of screening and participation rates among referral strategies and to determine whether screening and participation rates differed according to (1) ATF versus no ATF and (2) POC versus letter. We applied a one-sample test of proportion (in the case of comparing proportions) to examine representativeness in terms of demographic characteristics of the enrolled participants compared with census data. We further conducted group comparisons using one-way analysis of variance tests for continuous variables and chi-square tests for categorical variables. Two-tailed *P* values <.05 were considered statistically significant for this study.

Costs of recruitment were prospectively and retrospectively estimated based on the costs of recruitment materials (eg, handouts or flyers), supplies, and recruitment activities, including telephone follow-up, telephone screening, and in-person sessions. Labor costs were calculated using the research assistant (RA) annual salary (US $25,000) and publicly available average salary estimates for primary care physicians (US $187,013) and clinic managers (US $65,356). RAs tracked the time spent on various recruitment activities, number of phone calls made, enrollment sessions completed, and number of flyers printed in a custom computer database. Cost results are presented for each referral strategy.

## Results

### Reach

Over a period of 16 weeks, 2534 eligible patients visited the clinic. The maximum number of referrals that could have been made during well or chronic care visits over this time were approximately 30% of patient visits (n=991; hereafter referred to as potential referrals). The actual number of referrals made by the 5 physicians was 573 (274, 47.8% women; average age 55.7, SD 16.8 years) and, out of the 573 referrals, 98 (17.1%) patients were enrolled, representing an overall enrollment rate of 10% of the potentially eligible patient population. Of the 485 potential letter referrals, 485 (100%; 46% women; average age 56.3 years) were completed, 229 (47.2%; 48% women; average age 56.1 years) patients were screened by telephone, and 57 (11.8%; 58% women; average age 55.8 years) were enrolled. Of the 506 potential POC referrals, 88 (17.4%; 56% women; average age 52.0 years) patients were referred, 60 (11.9%; 57% women; average age 51.6 years) were screened, and 41 (8.1%; 61% women; average age 47.6 years) were enrolled. Patients receiving ATF were more likely to be screened (49% vs 7%; *P<*.001) and enrolled (15% vs 7%; *P<*.001) than those without ATF. Chi-square test results revealed significant differences in terms of patient screening (χ^2^_3_[573]=238.6; *P<*.001) and enrollment status (χ^2^_3_[573]=69.2; *P<*.001) among referral strategies (Table 2). Specifically, based on the number of referrals completed, there were variations in the proportion and absolute number of enrollees among the four referrals strategies (POC with ATF: 27/190, 50%; POC no ATF: 14/316, 41%; letter ATF: 30/199; 15.1%; letter no ATF: 26/286, 9.1%). Table 2 outlines the number and proportion of potential referrals, referrals made, patients screened, and patients enrolled.

**Table 2:** Reach results by referral strategy (N=991).

Strategy	Potential referrals	Referrals made, n (%)	Patients screened, n (%)	Patients screened and eligible, n (%)	Patients enrolled, n (%)
POC^a^+ATF^b^	190	54 (28.4)	45 (23.7)^c,d,e^	27 (14.2)	27 (14.2)^c^
POC	316	34 (10.8)	14 (4.4)^d,e,f^	14 (4.4)	14 (4.4)^d,e,f^
Letter+ATF	199	199 (100)	147 (73.9)^c,e,f^	30 (15.1)	30 (15.1)^c^
Letter	286	286 (100)	30 (10.5)^c,d,f^	27 (9.4)	26 (9.1)^c^
Total	991	573 (57.8)	236 (23.8)	98 (9.9)	97 (9.8)

^a^POC: point of care.

^b^ATF: active telephone follow-up.

^c^Significantly different from POC at α=.05.

^d^Significantly different from letter+ at α=.05.

^e^Significantly different from letter at α=.05.

^f^Significantly different from POC+ at α=.05.

Tables 3 and 4 present the demographic characteristics of participants and the target population and include comparisons of those characteristics among the referral strategies. Among those who enrolled, 59% were women, had an average age of 52.3 (SD 14.3) years, and an average BMI of 35.5 (SD 7.5) kg/m^2^. When compared with the target population (Butler County, Nebraska), participants did not significantly differ among demographic characteristics. Comparing by referral strategies, participants differed significantly in age.

**Table 3:** Representativeness of study sample (N=97).

Demographics	Target population: Butler County, NE^a^	Total enrolled participants
Age, median (years)	43.3	55.0^b^
Sex (female), n (%)	4026 (50.1)	58 (59)
Obese at baseline, %	(32)^c^	75 (77)^b^
White, n (%)	7846 (97.7)	91 (94)
Hispanic or Latino, n (%)	287 (3.6)	3 (3)

^a^NE: Nebraska; data as per census.gov 2019 estimates.

^b^Significant difference between target population and sample at α=.05.

^c^Only percentage estimate available.

**Table 4:** Representativeness comparisons among participants by referral strategies (N=97).

Demographics	Total sample	POC^a^+ATF^b^ (n=27)	POC no ATF (n=14)	Letter+ATF (n=30)	Letter no ATF (n=26)	Chi-square (*df*)	ANOVA^c^ (*df*)	*P* value
Age (years), mean (SD)	52.3 (14.3)	46.0 (14.7)^d^	51.9 (9.3)	58.7^e^ (13.1)	53.4 (15.1)	N/A^f^	*F*_93_=4.1 (3)	.009
Sex (female), n (%)	58.8	59	64	57	58	0.2 (3)	N/A	.97
Baseline BMI mean (SD), kg/m^2^	35.5 (7.5)	37.1 (6.8)	38.1 (9.1)	38.1 (9.1)	33.2 (6.6)	N/A	*F*_72_=1.5 (3)	.22
Age >65, n (%)	23 (23.7)	7.4^d^	7.1	43.3^e^	26.9	12.6 (3)	N/A	.07
White individuals, n (%)	94	93	93	100	92	2.3 (3)	N/A	.51
Hispanic or Latino, n (%)	3	3.7	0	0	7.7	3.1 (3)	N/A	.37

^a^POC: point of care.

^b^ATF: active telephone follow-up.

^c^ANOVA: analysis of variance.

^d^Significantly different from letter+ATF at α=.05.

^e^Significantly different from POC+ATF at α=.05.

^f^N/A: not applicable.

### Costs

Table 5 provides an overview and categorization of costs across referral strategies. Costs were determined based on nonlabor costs (US $738) and labor costs (US $5380), which were summed to provide the overall costs of recruitment (US $6118). Table 6 provides costs by referral strategy. All reported costs are rounded to the nearest dollar. We estimated an average of 2 minutes per POC referral based on anecdotal reports from the physicians; therefore, costs related to labor for POC referrals were estimated at US $162 for POC with ATF and US $102 for POC without ATF. Labor costs related to RA time spent making recruitment calls were the highest single line item costs and varied greatly between ATF referrals (US $1788) and referrals without ATF (US $135). The varying labor and nonlabor costs yielded different costs per enrolled participant via each referral strategy: letters with ATF was the costliest, at US $86 per enrolled participant, whereas POC with ATF (US $50), POC without ATF (US $61), and letter without ATF (US $51) costs were comparable.

**Table 5:** Breakdown of recruitment costs, rounded to the nearest dollar.

Cost element		Cost (US $)
**Nonlabor**		
	Printing letters and program descriptions, n=970	43
	Postage	267
	Envelopes, n=485	58
	POC cards	170
	IT^a^ (phone line)	200
**Labor**		
	Physician time spent making referrals	264
	Clinic manager time spent pulling patient list, 2 hours	63
**Research assistant time**		
	Training clinical staff, 2 hours	48
	Letter preparation, 24 hours	577
	POC^b^ preparation, 8 hours	192
	Recruitment calls, 80 hours	1923
	Enrollment visit preparation, 4 hours	96
	Enrollment visit, 49 hours	1178
	Mileage reimbursement to enrollment visits	1039
Total recruitment costs		6118
Total recruitment costs per enrolled participant		63

^a^IT: information technology.

^b^POC: point of contact.

**Table 6:** Recruitment costs in terms of yield by referral strategy, rounded to the nearest dollar (N=98).

Cost element			POC^a^+ (US $; n=27)		POC (US $; n=14)		Letter+ (US $; n=30)		Letter (US $; n=27)		Total sample (US $)
**Nonlabor**											
	Printing letters and program descriptions, n=970	N/A^b^		N/A		18		25		43	
	Postage	N/A		N/A		110		157		267	
	Envelopes, n=485	N/A		N/A		24		34		58	
	POC cards	99		71		N/A		N/A		170	
	IT^c,d^ (phone line)	50		50		50		50		200	
**Labor**											
	Physician time spent making referrals	162		102		0		0		264	
	Clinic manager time spent pulling patient list, 2 hours	0		0		31.50		31.50		63	
**Research assistant time**											
	Training clinical staff, 2 hours^d^	12		12		12		12		48	
	Letter preparation, 24 hours	N/A		N/A		237		340		577	
	POC preparation, 8 hours	73		119		N/A		N/A		192	
	Recruitment calls, 80 hours	346		58		1442		77		1923	
	Enrollment visit prep, 4 hours	24		24		24		24		96	
	Enrollment visit, 49 hours	324		165		365		324		1178	
	Mileage reimbursement to enrollment visits^d^	259.75		259.75		259.75		259.75		1039	
Total recruitment costs			1350		861		2573		1334		6118
Total recruitment costs per enrolled participant			50		61		86		51		63

^a^POC: point of contact.

^b^N/A: not applicable.

^c^IT: information technology.

^d^These items or activities were not specific to any one referral strategy; therefore, their costs were split evenly among each of the referral strategies.

## Discussion

### Principal Findings

Our results provided some support for the hypothesis that a physician letter referral with ATF is most effective for enrolling a large number of participants into a digitally delivered weight management program compared with POC referrals with and without telephone follow-up and physician letter referrals without follow-up. However, when considering the combined outcomes of penetration into the target population, overall and proportional yield, and cost of recruitment, letter referral without ATF may be the most attractive option for rural primary care clinics that wish to recruit patients into a weight management program. Importantly, all (100%) of the 485 potential letter referrals were made, as compared with the 88 (17%) of the 506 potential POC referrals. Letter with ATF (n=30), letter without ATF (n=27), and POC with ATF (n=27) yielded a similar number of participants. Finally, the costs associated with the letter without ATF referral strategy were relatively low (US $51 per enrolled participant).

Owing to the financial constraints of this pilot study, we limited the number of potential referrals to 991, knowing that this would be far less than the total number of eligible patients seen by the 5 physicians during the recruitment period. Awareness of the finite number of referrals may have influenced the physicians’ decision to refer a patient to the program, perhaps causing them to pass on referring an eligible patient to *save* that referral for a patient in greater need. However, we do not believe this influence was significant because not all of those potential referrals were used; <60% (573) of the potential referrals were distributed to eligible patients. Although not assessed in this study, primary care physicians often cite a lack of time during patient visits as a barrier to health counseling [26,27], which may also be the reason why the referral distribution rate was low among POC referrals. In addition, it is possible that a physician attempted to refer a patient during a clinic visit but the patient immediately declined the referral to the program. We were unable to quantitatively track this, which may have caused our POC distribution rate to reflect an underrepresentation of reality.

We calculated the proportional yield per referral strategy using potential referrals and referrals made as the denominator. These two analyses revealed different stories. When considering enrollees compared with potential referrals, the letter with active follow-up (15%) and POC with active follow-up (14%) strategies appear to be the best for enrolling a large number of participants into the weight loss program. However, when considering enrollees compared with the number of referrals made, POC with active follow-up (50%) emerges as the clear leader for proportional yield. When considering costs, POC with ATF again appeals as the least expensive recruitment strategy (US $50 per participant), although letter referrals without ATF (US $51) had similar costs. The time-intensive nature of recruitment strategies with ATF not only drives up costs but also places a higher burden on recruitment personnel compared with strategies without ATF. In kind, the cumulative time associated with POC referrals may be similar to that of clinic administrator time spent preparing the letter referrals. Primary care clinics that are considering referring patients to a weight management program should reflect on these approaches with regard to clinical flow and costs. Resource costs in the large volume of letters and telephone calls for letter referral with ATF may make POC referral strategies with active follow-up appealing to small rural clinics, or if system processes are in place to make patient identification and mailing easy, the letter referral without ATF may be the most attractive.

Similar to previous studies [5,11], our findings supported our hypothesis that referrals with ATF would yield a higher proportion of enrollees from those who received a referral. Conversely, our results did not match prior literature [5] and did not support our hypothesis that ATF would yield a lower absolute number of participants. The two conditions with ATF yielded the highest absolute number of participants (letter with ATF, 30 participants; POC with ATF, 27 participants), whereas the letter-only condition yield was comparable (26 participants). Our results supported our hypothesis that POC referrals would have a higher proportional yield of enrolled participants from those who received a referral compared with those who received a letter referral. The behavioral intervention literature has not definitively ruled on a dominant recruitment strategy [10,15]. Our findings provide valuable insight into the reach, representativeness, and cost of POC referrals and electronic health record registry–derived referral letters with and without ATF.

Our sample was older and had a higher proportion of females than the target population. This is easily explained by the following: (1) our intervention was limited to adults aged ≥19 years, whereas the male age of Butler County represents children and adults; (2) women traditionally have higher health care use than men [28]; and (3) excluding infants aged <1 year, older adults aged ≥65 have the highest physician rate by age group [29]. Our sample did not differ on any other demographic variable that was measured, which aligns with representativeness comparisons of active and passive recruitment strategies made by Lee et al [14] but contrasts with results reported by Linnan et al [12], who found that passive strategies yielded lower enrollment but a more diverse and higher-risk sample. Furthermore, among the four referral strategies, those with ATF had the highest (58.7 years, letter with ATF) and lowest (46.0 years, POC with ATF) mean age among participants, therefore providing no support for our hypothesis that ATF strategies would yield a more representative sample than referral strategies without ATF. Although not a significant difference, baseline BMI among enrollees from both POC referral strategies was higher than both letter referral conditions. This may point to an unintentional bias by physicians to refer patients with much higher BMIs than is required for eligibility. Interestingly, POC strategies were more successful at enrolling younger patients (<65 years) than the letter strategies. Although the literature is scant on this specific outcome and drawing any generalizable conclusions from this finding is unjustified, it nonetheless provides an intriguing area for further investigation.

Identifying the best method for maximizing the reach of a weight management program is important for future scale-up efforts. However, maximizing participant retention is a critical next step in the scale-up of any behavioral intervention. An interesting future point of investigation will be the intervention attrition gap of our intervention—the proportion of patients who initially enroll in the program and subsequently do not engage with any program features. This is an especially curious investigation given the conflicting literature regarding the effectiveness of active versus passive recruitment strategies for retaining participants in behavioral interventions [12,13].

There are limitations to our study that should be considered when interpreting our results. As the selected intervention and referral methods were tailored to fit the needs of our target population, the findings of our feasibility study may not be fully generalizable to other primary care clinics. However, the process by which we selected an evidence-based intervention is likely translatable to other researchers and practitioners attempting to select and implement an evidence-based intervention. As previously mentioned, we were not able to track the number of times a physician attempted to make a referral during a clinic visit but was immediately rejected by the patient being seen. Anecdotally, we can attest that this was a rare occurrence, as the physicians reported that the majority of patients were willing to receive or inquire for more information about the program. We did not collect demographic or qualitative data from the 5 physicians involved in referrals and therefore cannot comment on their national representativeness to physicians caring for rural patients or their perceived lack of time for distributing referrals. In addition, census data used to compare our sample with the target population were drawn from Butler County, Nebraska. However, the clinic from which we recruited participants may draw patients from outside Butler County, and comparisons may not directly align. However, when our sample is compared with the demographic characteristics of all adjacent counties (9 total), it remains a representative sample. Indeed, our study has several strengths. Our sample was 59% female, which is a higher proportion of male participants than typical community weight loss programs [30]. Our sample was representative of the racial and ethnic characteristics of the region. Although this feasibility study is small in scale, it addresses key areas of translational research as defined by RE-AIM [31-33] as well as areas of focus cited as integral to feasibility studies, such as adaptation, acceptability, practicality, and integration [34].

### Conclusions

We conclude that primary care physicians serving a rural community are capable of referring patients to a digitally delivered behavioral weight management intervention through in-person and population health record–generated letter referrals. When compared with POC methods, letter referrals were more effective at achieving high penetration in the target population and had a slightly higher yield of enrolled patients in this study. However, POC referrals yielded a considerably higher proportion of enrollees from those who received a referral. Participants who received ATF were more likely to be screened and enrolled in the program. These results suggest that either a letter or POC referral strategy can be effective with ATF; however, when resource costs are considered, POC with ATF may be the best method to engage rural primary care patients in a weight management program.

## Abbreviations

ATF: active telephone follow-up
POC: point of care
RA: research assistant
RE-AIM: Reach, Effectiveness, Adoption, Implementation, and Maintenance
